# Phenotypic Diversity and Plasticity of Photoresponse Across an Environmentally Contrasting Family of Phytoflagellates

**DOI:** 10.3389/fpls.2021.707541

**Published:** 2021-08-25

**Authors:** Mark R. Clegg, Alexander Wacker, Elly Spijkerman

**Affiliations:** ^1^Department of Ecology and Ecosystem Modelling, Institute of Biochemistry and Biology, University of Potsdam, Potsdam, Germany; ^2^Department of Theoretical Aquatic Ecology and Ecophysiology, Institute of Biochemistry and Biology, University of Potsdam, Potsdam, Germany; ^3^Animal Ecology Group, Zoological Institute and Museum, University of Greifswald, Greifswald, Germany

**Keywords:** photoresponse, behaviour, physiology, composition, photosynthesis, acclimation, *Chlamydomonas*, ecophysiology

## Abstract

Organisms often employ ecophysiological strategies to exploit environmental conditions and ensure bio-energetic success. However, the many complexities involved in the differential expression and flexibility of these strategies are rarely fully understood. Therefore, for the first time, using a three-part cross-disciplinary laboratory experimental analysis, we investigated the diversity and plasticity of photoresponsive traits employed by one family of environmentally contrasting, ecologically important phytoflagellates. The results demonstrated an extensive inter-species phenotypic diversity of behavioural, physiological, and compositional photoresponse across the Chlamydomonadaceae, and a multifaceted intra-species phenotypic plasticity, involving a broad range of beneficial photoacclimation strategies, often attributable to environmental predisposition and phylogenetic differentiation. Deceptively diverse and sophisticated strong (population and individual cell) behavioural photoresponses were observed, with divergence from a general preference for low light (and flexibility) dictated by intra-familial differences in typical habitat (salinity and trophy) and phylogeny. Notably, contrasting lower, narrow, and flexible compared with higher, broad, and stable preferences were observed in freshwater vs. brackish and marine species. Complex diversity and plasticity in physiological and compositional photoresponses were also discovered. Metabolic characteristics (such as growth rates, respiratory costs and photosynthetic capacity, efficiency, compensation and saturation points) varied elaborately with species, typical habitat (often varying more in eutrophic species, such as *Chlamydomonas reinhardtii*), and culture irradiance (adjusting to optimise energy acquisition and suggesting some propensity for low light). Considerable variations in intracellular pigment and biochemical composition were also recorded. Photosynthetic and accessory pigments (such as chlorophyll *a*, xanthophyll-cycle components, chlorophyll *a*:*b* and chlorophyll *a*:carotenoid ratios, fatty acid content and saturation ratios) varied with phylogeny and typical habitat (to attune photosystem ratios in different trophic conditions and to optimise shade adaptation, photoprotection, and thylakoid architecture, particularly in freshwater environments), and changed with irradiance (as reaction and harvesting centres adjusted to modulate absorption and quantum yield). The complex, concomitant nature of the results also advocated an integrative approach in future investigations. Overall, these nuanced, diverse, and flexible photoresponsive traits will greatly contribute to the functional ecology of these organisms, addressing environmental heterogeneity and potentially shaping individual fitness, spatial and temporal distribution, prevalence, and ecosystem dynamics.

## Introduction

Organisms often employ numerous and varied ecophysiological strategies to exploit environmental conditions and ensure ecological success (e.g., Grémillet et al., 2005; Clegg et al., 2012). Such diversity and “phenotypic plasticity” (rapid, reversible adjustment of an organism's observable traits) can maximise ecological fitness in fluctuating environments (e.g., West-Eberhard, 1989). However, the complexities involved are not always fully understood (Miner et al., 2005) and have rarely been comprehensively measured across a family of organisms. Therefore, in this study, we investigate the diversity and plasticity of ecophysiological (behavioural, physiological, and compositional) responses potentially employed by one family of green algal (chlorophyte) phytoflagellates, the Chlamydomonadaceae.

Phytoflagellates are an ecologically important group of eukaryotic microorganisms whose dual motility and photosynthesis bridge the divide between animal and plant ecophysiology. They are integral and thrive in a wide range of habitats; and they have recently assumed increased relevance due to their role in harmful algal blooms and biofuel synthesis (e.g., Smayda, 1997; Benning, 2015). Chlamydomonadaceae are most abundant in freshwaters: yet they occur in virtually every aquatic ecosystem (spanning the trophic continuum) and in terrestrial (aqueous interstitial), snow, ice, soil and, rocky substrate environments (e.g., Ettl, 1983). However, although widely used in biomolecular investigations (e.g., Merchant et al., 2007), their ecology still remains uncertain, and the ecophysiological predisposition and differentiation of environmentally contrasting species have rarely been described.

All phytoflagellates are strongly influenced by a wide range of environmental factors that affect growth, survival, and distribution (e.g., Clegg et al., 2007; Mellard et al., 2012). The availability of light is especially important, particularly for the photoautotrophic Chlamydomonadaceae, as temporal and spatial heterogeneity in irradiance will influence photosynthesis (e.g., Falkowski and Raven, 2007) and may lead to an expression of various photobiological traits that could overcome light limitation (e.g., Litchman and Klausmeier, 2008; Clegg et al., 2012; Suggett et al., 2015).

Motile Chlamydomonadaceae are able to tackle the intricacies of their photic environment *via* an active behavioural response, involving sensory photodetection and controlled swimming to favourable conditions. Sensory-mediated locomotory photoresponse has been extensively studied in phytoflagellates (from Engelmann, 1882 to Coesel et al., 2021), but less frequently from an ecological perspective, despite its potential importance. It is generally the hierarchically dominant behaviour (Clegg et al., 2004b) and may energetically optimise growth (Clegg et al., 2003), the acquisition of resources (e.g., Heaney and Eppley, 1981), and contribute to spatial and temporal distribution (e.g., Clegg et al., 2007). Interestingly, previous investigations have observed an unusual preference for relatively low light in two species of *Chlamydomonas* (Clegg et al., 2003, 2012). This paradoxical preference could be a familial trait; it may be linked to other behavioural strategies (Clegg et al., 2007), it may act as a surrogate signal for the greater availability of nutrients at depth, or it may be ecophysiologically motivated (Clegg et al., 2012).

Physiological and compositional photoresponses in the Chlamydomonadaceae have also been widely studied, but again ecological aspects of their diversity remain unknown. Metabolic energy acquisition in microalgae can vary considerably with species and irradiance (e.g., Richardson et al., 1983; Ajani et al., 2021). Physiological alterations in light harvesting capacity and efficiency can optimise photosynthesis (e.g., Falkowski and Raven, 2007) and are often driven by dynamic compositional reorganisation of the photosynthetic architecture. Indeed, stoichiometric diversity of intracellular pigment and fatty acid composition (with species and environment) can energetically exploit the quantity and quality of ambient light (e.g., Falkowski and Raven, 2007; Stomp et al., 2008).

In addition to this possible diversity, phytoflagellate Chlamydomonadaceae could also photobiologically adjust to variations in light, as differentiated in Falkowski and Laroche (1991), *via* evolutionary, longer term “photoadaptation” (responsible for creating differences between taxa), and/or by phenotypically expressed, rapid, and readily reversible “photoacclimation” (through behavioural, physiological, and compositional plasticity).

Studies on behavioural photoacclimation in phytoflagellates are very rare. However, limited plasticity has been observed in larger, more evolutionary complex (ecological *K*-type) species, such as *Ceratium furcoides* (Clegg et al., 2003), and may augment vertical positioning and migration. Physiological and compositional photoacclimation strategies have been observed more widely (e.g., Falkowski and Chen, 2003; Wacker et al., 2016) and, as noted, can include complex taxon-specific alterations in the efficiency, capacity, and intracellular architecture of photosynthesis (e.g., Falkowski and Raven, 2007; Suggett et al., 2015). In chlorophytes, for example, characteristic increases in antennal chlorophyll that optimise energy acquisition at low light may be intricately linked to the redox poise of electron transport pathways (e.g., Falkowski and Chen, 2003).

Flexible phenotypic photoacclimation (particularly in response to low light) is of functional and ecological importance (Richardson et al., 1983; Litchman and Klausmeier, 2008), and because there remain appreciable gaps in the understanding of such strategies in phytoflagellates, there is a compelling need for ecological-based comparisons.

To the knowledge of the authors, no studies have used an expansive interdisciplinary approach to examine the potential expression and flexibility of ecophysiological photoresponse across a family of organisms. Therefore, the primary aim of this study was to investigate both the general diversity and the plasticity of behavioural, physiological, and compositional responses to ecologically representative quantities of light (and low light in particular), for the first time, across an environmentally and phylogenetically contrasting family of phytoflagellates. During this investigation, we sought to determine the occurrence, nature, and extent of these photoresponses across 11 species of Chlamydomonadaceae; analysing their degree of flexibility and possible environmental derivation, to help improve the understanding of their potential contribution to prevalence and survival in the environment, and to help resolve their broader functional ecophysiological and ecological importance.

## Materials and Methods

### Study Species and Typical Habitats

Eleven species of Chlamydomonadaceae, obtained from the SAG Culture Collection of Algae (Universität Göttingen, Germany), are used in this study and listed, with strain details, in Table 1. These phytoflagellates were selected based on their distinctive phylogeny but primarily on the contrasting type, salinity, and trophic nature of their typical ecological habitat. They included two ecotypes (snow/ice and brackish) of *Chlamydomonas augustae* Skuja, the mt+, ♂, isogamete of *C. moewusii* Gerloff, and the “standard strain” of *C. reinhardtii* Dangeard (designated as the conserved epitype in many phylogenetic studies). Microalgal taxonomy is often fluid, and although a couple of these species have been re-classified within the family since this investigation commenced, for simplicity of comparison we will follow their original nomenclature throughout. The phylogentic clade of each species was assigned according to the latest taxonomic consensus (e.g., Pröschold et al., 2001). In total, there were eight freshwater, two brackish, and one marine study species. The “typical recorded habitat” of each (noted in Table 1) was an average derived from a combination of the conditions found at the original isolation site and from sites of their natural occurrence (previously recorded in all publications, taxonomic sources, and algal identification guides that have attempted classification to a species level).

**Table 1:** The eleven contrasting species of *Chlamydomonas* used in this study.

**Species**	**Symbol**	**SAG strain**	**Original isolation date and site**	**Typical recorded habitat:**			**Phylogenetic clade**
				**Water type**	**Details**	**Nutrient status**	
*C. augustae*	*	26.86	1968 - on snow, Cascade Mountains, OR, USA	(freshwater)	snow + ice	oligotrophic	Chloromonas
*C. asymmetrica*	*	11-7	1952 - Lake Siggeforasjön, Sweden	freshwater	humic/dystrophic	oligotrophic	Reinhardtii
*C. chlorastera*	△	53.90	<1988 - Broa Reservoir, Brazil	freshwater	–	oligo-/mesotrophic	Chloromonas
*C. moewusii*	△	20.90	1951 - forest drainage ditch, Spandau, Germany	freshwater	–	oligo-/meso-/eutrophic	Moewusii
*C. orbicularis*	○	11-19	1930 - sandy shore of River Elbe, Czech Republic	freshwater	often fluvial	eutrophic	Reinhardtii
*C. reinhardtii*	○	11-32b	1945 - soil, near Amherst, MA, USA	freshwater	often soil	eutrophic	Reinhardtii
*C. segnis*	○	1.79	1969 - Delta Marsh, Manitoba, Canada	freshwater	–	eutrophic	Oogamochlamys
*C. monadina*	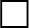	54.91	1984 - Priest Pot, Cumbria, UK	freshwater	–	eu-/hypertrophic	Monadina
*C. augustae* (br)	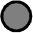	13.89	<1986 - brackish salt marsh pool, UK	brackish	–	usually eutrophic	Chloromonas
*C. uva-maris*	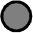	19.89	<1987 - brackish river, Wolferton, Norfolk, UK	brackish	–	usually eutrophic	Monadina
*C. parkeae*	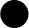	24.89	1962 - marine, the high seas, Plymouth, UK	marine	–	meso-/eutrophic	Moewusii

*Information includes the symbol notation of species (used throughout figures), culture strain details, and aspects of their typical recorded habitats (with each species primarily arranged by the salinity of water in which they typically occur and then by increasing nutrient status, in the case of freshwater species)*.

### Culture Conditions, Light Acclimation, and Growth

All the study species were grown for several generations in semi-continuous batch culture in 300-ml Erlenmeyer flasks. The freshwater species were grown in a modified Woods Hole (WC) medium (Nichols, 1973) at pH 7. The marine species was grown in artificial enriched seawater medium, ESAW (Berges et al., 2001), and the brackish species in ½ ESAW (i.e., a 50% reduction of salt solutions I and II), all at pH 7.8. To determine the effects of light acclimation (and particularly the influence of low light), the species were grown at two different culture irradiances; in low light “LL” conditions of 30 ± 2 μmol photons m^−2^ s^−1^ (photosynthetically active radiation: PAR) to simulate conditions lower down in a substrate/water column; and in high light “HL” conditions of 354 ± 42 μmol photons m^−2^ s^−1^ to reflect moderate to high environmental light conditions (but not so high as to induce resource limitations *via* overly dense cultures). All the species were grown in full-spectrum light, under a 16:8 h light:dark regime. Illumination was provided by fluorescent lamps (TL-D 18 and 58W/930, Philips, Eindhoven, The Netherlands), and irradiance was verified in each vessel (throughout culture) using a 4π spherical quantum sensor (US-SQS, Walz, Effeltrich, Germany).

Both low light and high light cultures were grown for at least 4 weeks (comprising several generations), to allow sufficient time to acclimate. Culture vessels were housed in climate cabinets, with temperatures remaining stable at 20.4 ± 1.2°C, and flasks were mixed regularly to prevent wall growth. To ensure similar conditions within LL and HL treatments, the cultures were operated as (semi-continuous) turbidostats and diluted every second day to give an optical density of 0.05 at 750 nm (measured using a UV mini-1240 spectrophotometer, Shimadzu, Kyoto, Japan). Daily and average growth rates (μ) were calculated, assuming exponential growth. The cell densities of cultures (that ranged from 4.5 × 10^4^ to 1.4 × 10^6^ cells ml^−1^) were also measured with an automatic cell counter (CASY 1, Schärfe System, Reutlingen, Germany). Subsequently, at the required time of harvest (and always during the 16-h light phase of culture), several aliquots from the LL and HL cultures of each species were extracted simultaneously for use in behavioural, physiological, and compositional analyses.

### Behavioural Measurements

To investigate behavioural response to light, aliquots of live cells taken from low light and high light cultures of all the species were exposed to a gradient of photon irradiance in a laboratory preference chamber, comparable with that verified for ecological applicability in Clegg et al. (2003). As described in Clegg et al. (2012), this chamber comprised a glass microscope slide modified to form an enclosed cavity (55 mm long by 20 mm wide by 0.33 mm deep). It was secured on the stage of an inverted microscope (Axiovert 25, Zeiss, Jena, Germany), and a light gradient was produced along its length using a series of neutral density filters (LEE Filters, Burbank, CA, United States) fixed 30 mm above the chamber and beneath a 20 W halogen light source (Osram, Munich, Germany). This created six gradually increasing light zones, with average irradiances of 13, 24, 47, 102, 182, and 232 μmol photons m^−2^ s^−1^, quantified using a 2π sensor (LI-190, LI-COR Biosciences, Lincoln, NE, United States). Small fans adjacent to the chamber prevented the build-up of radiant heat and thermocouples (Testo, Alton, United Kingdom) fixed in each end, verified that internal temperatures remained constant over time at 21 ± 0.9°C.

Following the methods described in Clegg et al. (2003, 2012), culture suspension was injected into the chamber, and population response and individual cell behaviour were recorded. Population response, defined as the difference between initial and final cell distribution (after a saturated-response experiment duration of 35 min), was measured five times for each species and culture light treatment. Differences in distribution were assessed using a log linear model (McCullagh and Nelder, 1983) constructed within a GenStat (9th Edition, VSN International Ltd., Hemel Hempstead, United Kingdom) program, and the proportion of cells displaying a preference for each light zone was tested using a three-model nested *F*-test (Hurley, 1996); to assess within treatment (hereafter termed: “*F*-test”) and between treatment and species (“modified *F*- test”) variation. Tukey tests of pairwise differences subsequently distinguished the specific zones in which significant accumulations or decreases in cell concentration occurred. Additional measurements of the swimming behaviour of individual cells [including cell-track analysis of their direction, speed, and movement kinetics: following the methods described in Clegg et al. (2012)], also determined the factors driving population response and characterised any behaviour as phototactic, ortho- or klino-photokinetic [after Diehn et al. (1977)].

### Physiological Measurements

In addition to measurements of growth rates during culture, further analyses investigated the diversity and plasticity of physiological response to light. First, several 50 ml aliquots (or replicates) from low light and high light cultures of the 11 species were taken, concentrated by centrifugation (at 3,000 × g, for 5 min, at 20°C) to an optical density of 0.2 (at 750 nm), and dark adapted at 20°C for 30 min (to standardise physiological base-levels). Prior to physiological measurement, portions of each aliquot were removed for cell enumeration and measurement of cellular carbon. Carbon concentration was measured by filtering ~0.15 mg carbon of the algal suspension onto 25 mm, precombusted, glass fibre filters (GF/ F; Whatman, Maidstone United Kingdom), and quantified using an elemental analyzer (HighTOC, Elementar, Langelselbold, Germany).

Photosynthetic oxygen evolution was then measured (following methods described in Spijkerman and Wacker 2011) over a range of 11 actinic light intensities (0–1,500 μmol photons m^−2^ s^−1^) for a period of 20 min using a Clark electrode (Microelectrodes Inc., Bedford, NH, United States) in a light dispensation system (PLD 2, Topgallant Partners, LLC, Londonderry, NH, United States). Rates of oxygen evolution (P) were corrected for carbon concentration and fitted to the equation:

(1) $$\begin{array}{r} \text{P}={\text{P}}_{\text{max}}\left(1-{\text{e}}^{-\alpha \text{E}/\text{Pmax}}\right)+\text{E}\beta +{\text{R}}_{\text{d}} \end{array}$$

where E is the photon irradiance, P_max_ is the maximum gross oxygen production (giving an indication of photosynthetic capacity), R_d_ is the respiration rate in the dark and therefore R_d_:P_max_ is the proportion of respiratory losses to photosynthesis (an indication of basic metabolic costs), α is the initial slope of the photosynthesis-irradiance curve (an indication of photosynthetic efficiency at low light), and β is the slope at high irradiances that describes photoinhibition. Finally, the irradiance above which photosynthesis was possible, E_c_ (the light compensation point), was calculated by dividing R_d_ by α, and the irradiance at which photosynthesis becomes saturated, E_k_ (the light saturation point), was calculated by dividing P_max_ by α.

### Compositional Measurements

The diversity and plasticity of compositional responses to light were also investigated. Simultaneous to physiological measurements, 50 ml aliquots (i.e., paired samples) from cultures of all species and both light treatments were taken, and the mass-specific pigment and fatty acid composition were measured in relation to cellular carbon content.

For pigment determination, culture suspension taken directly from the light was filtered onto GF/F (Whatman, Maidstone, United Kingdom) filters, immediately frozen in liquid nitrogen and stored at −80°C. Prior to analysis, the frozen filters were macerated in 90% acetone (in a Mini Beadbeater) and after centrifugation (18,000 × *g*, 5 min, 4°C) the supernatants were analysed by high performance liquid chromatography (HPLC), following the methods of Gilmore and Yamamoto (1991) and using a Spherisorb ODS-1 column (5 μm, non-endcapped, 6% carbon, 250 × 4.6 mm, Nordantec GmbH, Bremerhaven, Germany). Absorbance was recorded with a PHD 601 multi-wavelength UV-VIS detector (GAT, Bremerhaven, Germany). Pigment concentrations, ratios, and the de-epoxidation state of the xanthophyll-cycle components were determined following the methods described in Gerloff-Elias et al. (2005a) to quantify photosynthetic light absorbance capacity, low-light/shade adaptation, and photoprotection.

Fatty acid composition was measured following methods described in Spijkerman and Wacker (2011). Samples were obtained from the aliquots of culture suspension by filtering ~0.5 mg of algal carbon onto GF/F (Whatman, Maidstone, United Kingdom) filters. The filters were stored at −25°C in a nitrogen atmosphere, in Teflon sealed glass tubes in 7 ml of dichloromethane-methanol (2:1 v/v). Lipids were extracted and transesterified into FA methyl esters and were subsequently identified and quantified by gas chromatography [as in Wacker and Weithoff (2009)]. All replicate samples were measured at least twice, with total and specific fatty acid concentrations determined and ratios of polyunsaturated to saturated fatty acids (PUFA:SFA) calculated (as they are often indicative of thylakoid development and photoacclimation).

Statistical analyses of all physiological and compositional data were conducted using the software R (R Development Core Team, version 3.02, www.r-project.org). Physiological and compositional measurements from more than three (and up to six) replicate samples were analysed for each species. Due to the paired nature of the samples, the potential influence of culture irradiance (LL v. HL) on all species combined, and on each individual species, was tested statistically using paired *t*-tests; with additional analyses testing any differences due to the salinity, habitat trophy, and phylogenetic clade of species. All physiological and compositional data were also plotted separately in scatterplots and compared spatially against a theoretical 1:1 (*x* = *y*) relationship, with linear regressions calculated to illustrate the potential differences between the LL and HL cultures. Positive or negative trends in the data were indicated by the position, slope, and intercept of the regression in relation to the 1:1 line, with the fit of the slope of the regression giving an indication of between-species variation.

## Results

### Behavioural Response, Diversity, and Acclimation

**Figure 1:**
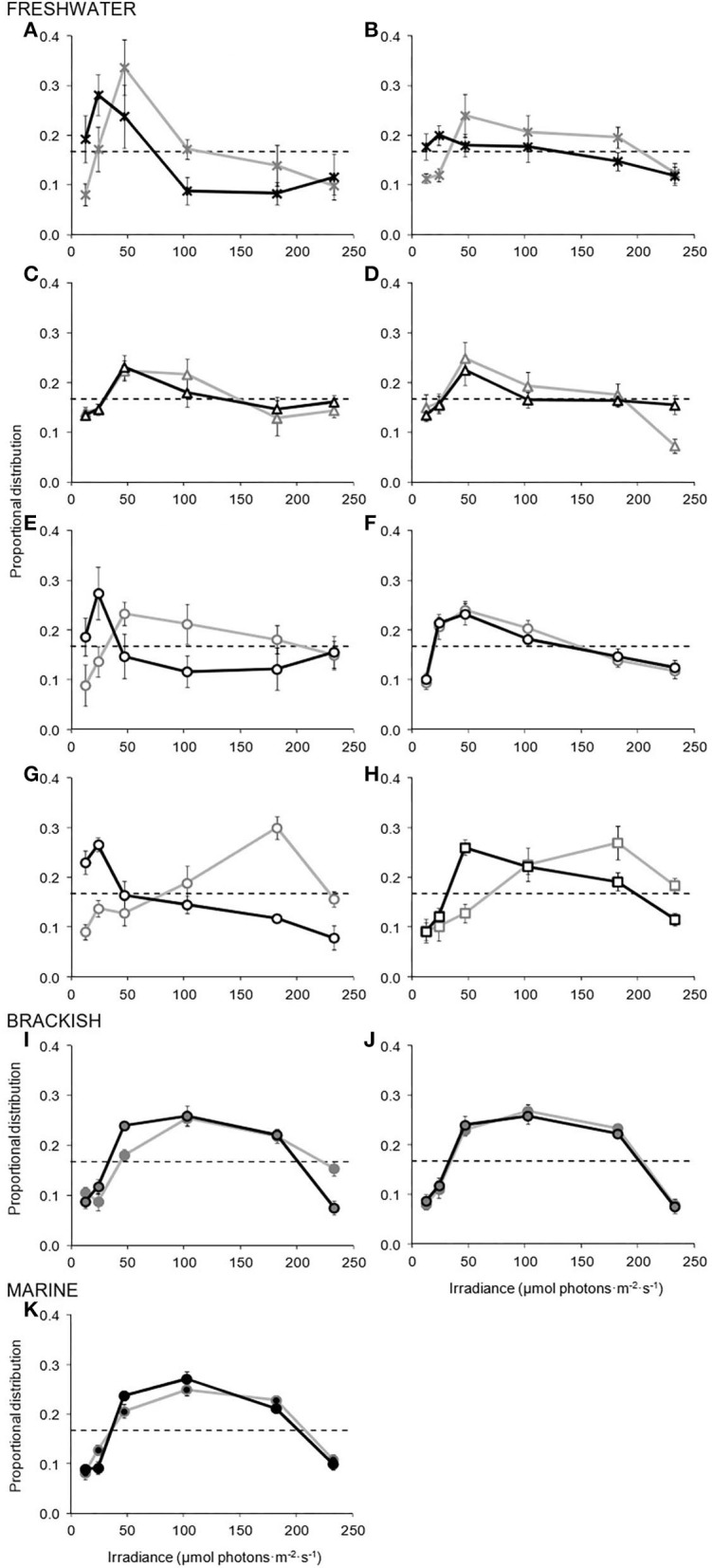
Population response curves showing the behavioural response of the 11 species of *Chlamydomonas* **(A–K)**, cultured at low light (LL, black curves and symbol outlines) and high light (HL, grey curves and symbol outlines), when exposed to a gradient of photon irradiance within a preference chamber. The spatial distributions of cells are presented as a proportion of 1 (with maximum likelihood estimates ± 2 SE of the mean). Dashed horizontal lines illustrate the homogeneous starting distribution (data points not shown) observed in both treatments. Graph panels are grouped according to the aquatic environments in which the species typically occur, with freshwater species also arranged *via* the increasingly nutrient-rich environments in which they are found. Symbol types and shading differentiate the contrasting trophy and salinity (respectively) of the aquatic environments in which each species is typically found, and are as presented in Table 1. **(A)** *C. augustae*. **(B)** *C. asymmetrica*. **(C)** *C. chlorastera*. **(D)** *C. moewusii*. **(E)** *C. orbicularis*. **(F)** *C. reinhardtii*. **(G)** *C. segnis*. **(H)** *C. monadina*. **(I)** *C. augustae* (br). **(J)** *C. uva-maris*. **(K)** *C. parkeae*.

Population response curves (Figures 1A–K) showed that in all 11 species, and at both culture irradiances (LL and HL), cells displayed a significant variation from initial homogeneous distributions (log-linear model *F*-test, *P* < 0.05, Table 2). All the species, therefore, exhibited a relatively strong behavioural response to light, with cells avoiding very low and high light, and accumulating at distinct, “preferred” irradiances. With the exception of the snow alga, *C. augustae*, there was generally an increase in the strength of response with an increase in the nutrient richness of the habitat of the species (concomitantly indicated by increased curve size and statistical significance, Figure 1, Table 2).

**Table 2:** Summary of predominant trends in the behavioural, physiological, and compositional data across the 11 species of *Chlamydomonas*.

**Species**	**Behaviour (** **Figures 1A–K** **):**		**Physiology (** **Figures 2A–F** **):**						**Composition (** **Figures 3A–E** **)**				
	**Preference (LL and HL cultures)**	**Adaptation**	**Growth**	**P**_**max**_	**R**_**d**_ **:P**_**max**_	**α**	**E**_**c**_	**E**_**k**_	**Chl *a***	**DPS**	**Chl *a*:*b***	**Chl *a*: carot**.	**PUFA:SFA**
*C. augustae*	L*** & L***	L↔L**	H*	(H)	(=)	(=)	(H)	H**	(L)	H**	H*	(=)	(L)
*C. asymmetrica*	L* & L*	L↔L**	H*	(H)	(=)	(L)	H*	H*	(L)	H**	(H)	(=)	(=)
*C. chlorastera*	L** & L**	(n.s.)	(H)	(=)	(=)	(L)	(H)	H**	(L)	(H)	(L)	L*	(L)
*C. moewusii*	L** & L**	(n.s.)	(H)	(H)	(=)	(L)	(H)	H*	L***	(=)	H*	(L)	(H)
*C. orbicularis*	L*** & **=** ***	**L↔=** **	(H)	(=)	(H)	L*	H**	H**	L**	(=)	(=)	(L)	(L)
*C. reinhardtii*	L** & L**	(n.s.)	H*	(H)	H*	L*	H***	H**	L*	H*	H***	L***	L*
*C. segnis*	L*** & H***	L↔H***	H*	(H)	(=)	(L)	(H)	(H)	L*	H*	(H)	(=)	(L)
*C. monadina*	L*** & H***	L↔H***	L*	(=)	(H)	(L)	H*	(H)	(L)	(H)	(H)	(L)	L*
*C. augustae* (br)	**=** *** & **=** ***	(n.s.)	H**	(L)	(H)	(L)	H*	(H)	(L)	(H)	(H)	L*	(=)
*C. uva-maris*	**=** *** & **=** ***	(n.s.)	H*	(L)	(H)	(L)	H**	(H)	(L)	(H)	(=)	L***	(=)
*C. parkeae*	**=** *** & **=** ***	(n.s.)	H**	(H)	(=)	(L)	H**	(H)	L**	(H)	(H)	(L)	L*

The direction of trends (derived from higher values in the data) are denoted by “L” for LL, “H” for HL, and “=” for LL = HL (or for moderate light in the case of behavioural preference). The statistical significance of these trends (derived from F-tests and paired t-tests) are also indicated (with significance shown using bold text); e.g., H^***^ = P < 0.001, H^**^ = P < 0.01, H^*^ = P < 0.05, (H) (L) (=) or (n.s.) = not significant.

Overall, there was a preference for relatively low levels of light in eight of the 11 species (all typically found in freshwater), although in some cases this preference was flexible. LL cultures of the freshwater species generally showed significant preferences for between 13–47 μmol photons m^−2^ s^−1^ (*F*-test, *P* < 0.05: Tukey test *P* < 0.05), although *C. monadina* (Figure 1H) showed a slightly higher preference for 47–102 μmol photons m^−2^ s^−1^ (*F*-test, *P* < 0.001: Tukey test *P* < 0.05). In contrast, LL and HL cultures of the marine and brackish species displayed a preference for higher light, 47–182 μmol photons m^−2^ s^−1^ (*F*-test, *P* < 0.001: Tukey test *P* < 0.05), significantly greater than the preferences of all freshwater LL and both LL and HL cultures of *C. chlorastera, C. moewusii*, and *C. reinhardtii* (modified log-linear model *F*-test, *P* < 0.01).

Complementary measurement of the swimming behaviour of individual cells re-enforced population response results and confirmed the nature of the photoresponse. Cell-track analyses showed that individual cells of all the species displayed significant directed orientation (Rayleigh's test of uniformity, *P* < 0.05) *via* positive and negative phototactic behaviour (Watson's *F*-test, *P* < 0.001), combined with significant photophobic reactions (increased percentage of cells turning) in the transition regions at light zone boundaries (ANOVA, *P* < 0.005; Tukey test, *P* < 0.05), to initially facilitate movement to and then maintain position within preferred light zones. Swimming speeds ranged from 54 ± 13 to 92 ± 21 μm s^−1^ across the species, and no ortho- or klino-photokinetic responses were observed (ANOVA, *P* > 0.1).

Five of the eight freshwater species demonstrated a significant increase in preference with an increase in culture irradiance (Table 2), indicative of an adaptation of behavioural population response. Generally, the strength of this adaptation increased with an increase in the trophic nature of the habitat of the species (Figure 1). *C. augustae*, for example, showed a slight increase in preference from 13–47 to 47 μmol photons m^−2^ s^−1^ (modified *F*-test, *P* < 0.01), *C. asymmetrica* and *C. orbicularis* showed slightly greater increases (modified *F*-test, *P* < 0.01), while *C. segnis* and *C. monadina* showed very large shifts in preference from low to high light with changing culture irradiance (modified *F*-test, *P* < 0.001). Six of the 11 species, including all brackish and marine species and the freshwater *C. chlorastera, C. moewusii*, and *C. reinhardtii*, showed no significant culture-light induced adaptation of behaviour (modified *F*-test, *P* > 0.05). Additionally, there was evidence of a slight progression of increasing behavioural preference from low to high light (in LL cultures) and degree of behavioural adaptation, with distance of separation on recent phylogenetic trees (e.g., Pröschold et al., 2001). The greatest genetic and behavioural differences were potentially between Oogamochlamys and Monadina clades, with a gradual increase in preference irradiance and reduction in behavioural adaptation from Oogamochlamys, Reinhardtii, Chloromonas, Moewusii through to Monadina clades.

### Physiological Diversity and Acclimation

**Figure 2:**
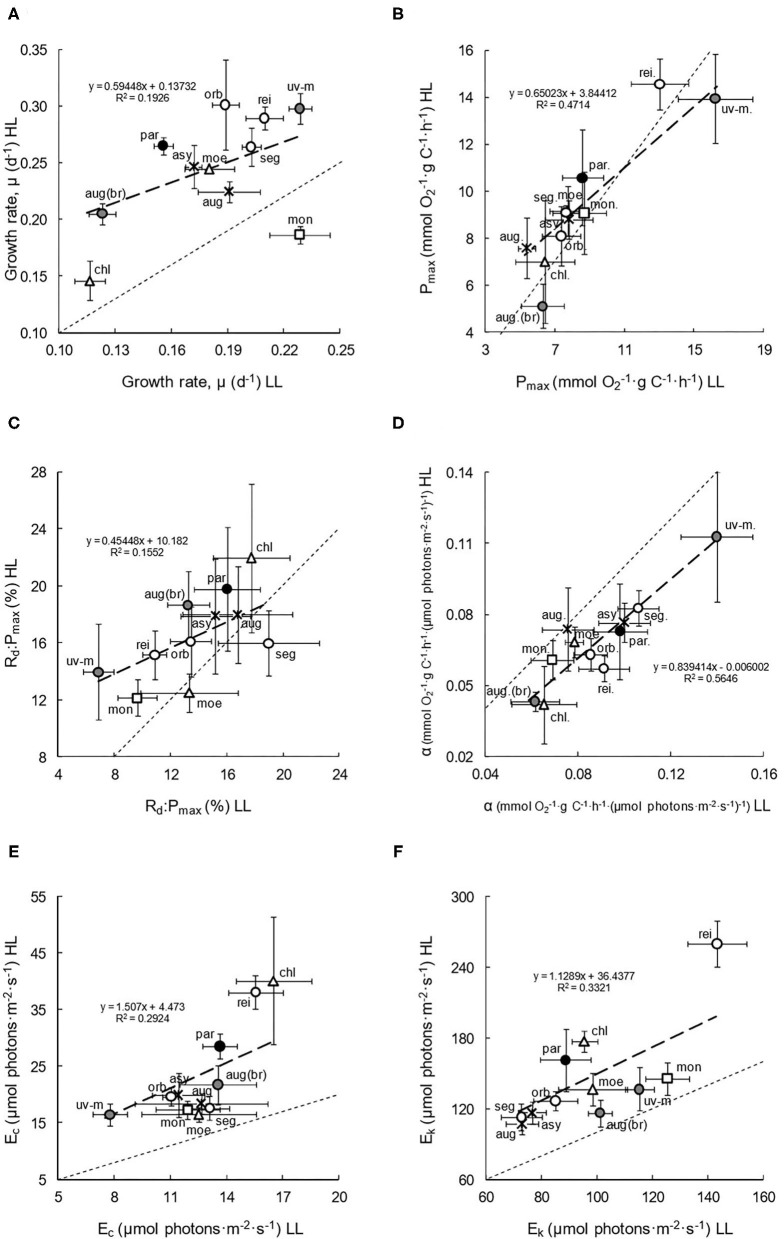
Physiological characteristics **(A–F)**, of the 11 species of *Chlamydomonas* grown under conditions of low light (LL) and high light (HL). Mean values and standard errors are presented in each scatterplot. The thin dashed lines represent theoretical 1:1 (*x* = *y*) relationships between LL and HL adapted cultures, with thicker, linear regressions describing the observed relationships. The spatial spread of species data points gives an indication of intra-familial diversity, and their position above or below the 1:1 line indicates higher values in HL or LL culture irradiances, respectively, thereby suggesting plasticity. Symbols and shading are as presented in Table 1. Species are abbreviated as follows: asy, *C. asymmetrica*; aug, *C. augustae*; aug (br), *C. augustae* (brackish); chl, *C. chlorastera*; moe, *C. moewusii*; mon, *C. monadina*; orb, *C. orbicularis*; par, *C. parkeae*; rei, *C reinhardtii*; seg, *C. segnis*; uv-m, *C. uva-maris*. **(A)** Growth rate. **(B)** P_max_. **(C)** R_d_:P_max_. **(D)** α. **(E)** E_c_. **(F)** E_k_.

The physiological results (presented in scatterplots and analysed statistically: Figures 2A–F, Table 2) demonstrated the diversity of growth, photosynthetic, and respiratory characteristics and the effect of irradiance across the 11 species. Growth rates (Figure 2A) typically increased with increasing culture irradiance (paired *t*-test for all species: df = 38, *P* < 0.001; Table 2 for individual species), except in *C. monadina*, with species usually positioned above the 1:1, *x* = *y* (LL = HL) axis relationship line in the scatterplot (a trend that is confirmed by the position of the linear regression). The greatest increase was in brackish, marine, and eutrophic species. Irrespective of culture light, the eutrophic species also generally showed higher rates of growth, while members of the Chloromonas clade showed slightly lower growth.

Photosynthetic capacity (P_max_) in relation to carbon content (Figure 2B) generally showed no significant acclimation to light (paired *t*-test: df = 50, *P* = 0.168; Table 2), with both the data and regression overlapping the 1:1 line in the scatterplot. It did not vary significantly with species (paired *t*-tests: dfs = 2 to 5, due to different replicate numbers of species; *P* > 0.06). However, the brackish species did show a slight increase in P_max_ in LL and, on average, the P_max_ of both LL and HL cultures may be slightly higher in species found in more nutrient-rich habitats. R_d_:P_max_ (Figure 2C), a basic indication of metabolic costs, was slightly but significantly higher in HL cultures (paired *t*-test: df = 50, *P* < 0.01), even though the regression did partially overlap the 1:1 line. On a species level, the increase in HL was only significant in *C. reinhardtii* (paired *t*-test: df = 5, *P* < 0.05: Table 2), although marine and brackish species also showed a large acclimation. Overall, metabolic costs may be slightly lower in most eutrophic species and in the Monadina clade.

Photosynthetic efficiency at low irradiance (α) in relation to carbon content (Figure 2D) was significantly higher in low light-adapted cultures (paired *t*-test: df = 50, *P* < 0.001: Table 2), with species positioned below the 1:1 line. The eutrophic species (e.g., *C. reinhardtii* and *C. orbicularis*: paired *t*-tests: dfs = 5 and 4, *P* < 0.05) and marine and brackish species tended to show the highest degree of LL acclimation (Figure 2D), and, overall, α was slightly lower in the Chloromonas clade.

Compensation points, E_c_ (Figure 2E), were significantly lower in the low light cultures (paired *t*-test: df = 50, *P* < 0.001: Table 2), with species therefore positioned above the 1:1 line. A central clustering of species indicated a generally consistent degree of acclimation, with no significant differences derived from salinity, habitat trophy, or clade. However, the greatest change in E_c_ with culture light occurred in *C. reinhardtii* and *C. parkeae*, and the highest and lowest overall E_c_ occurred in *C. chlorastera* and *C. uva-maris*, respectively. The saturation point for photosynthesis, E_k_ (Figure 2F) was also slightly and significantly lower in most of the LL cultures (paired *t*-test: df = 50, *P* < 0.001: Table 2), with species again positioned above the 1:1 line. In general, the lowest variation in E_k_ with culture light seemed to be in the brackish, marine, and hypertrophic species (Figure 2F, Table 2), and the highest was in the Reinhardtii clade, greatly skewed by the high E_k_ and large acclimation of *C. reinhardtii*. Overall, in most cases, species that showed adaptation of E_c_ tended to show lesser adaptation of E_k_, and vice-versa (Table 2).

### Compositional Diversity and Acclimation

**Figure 3:**
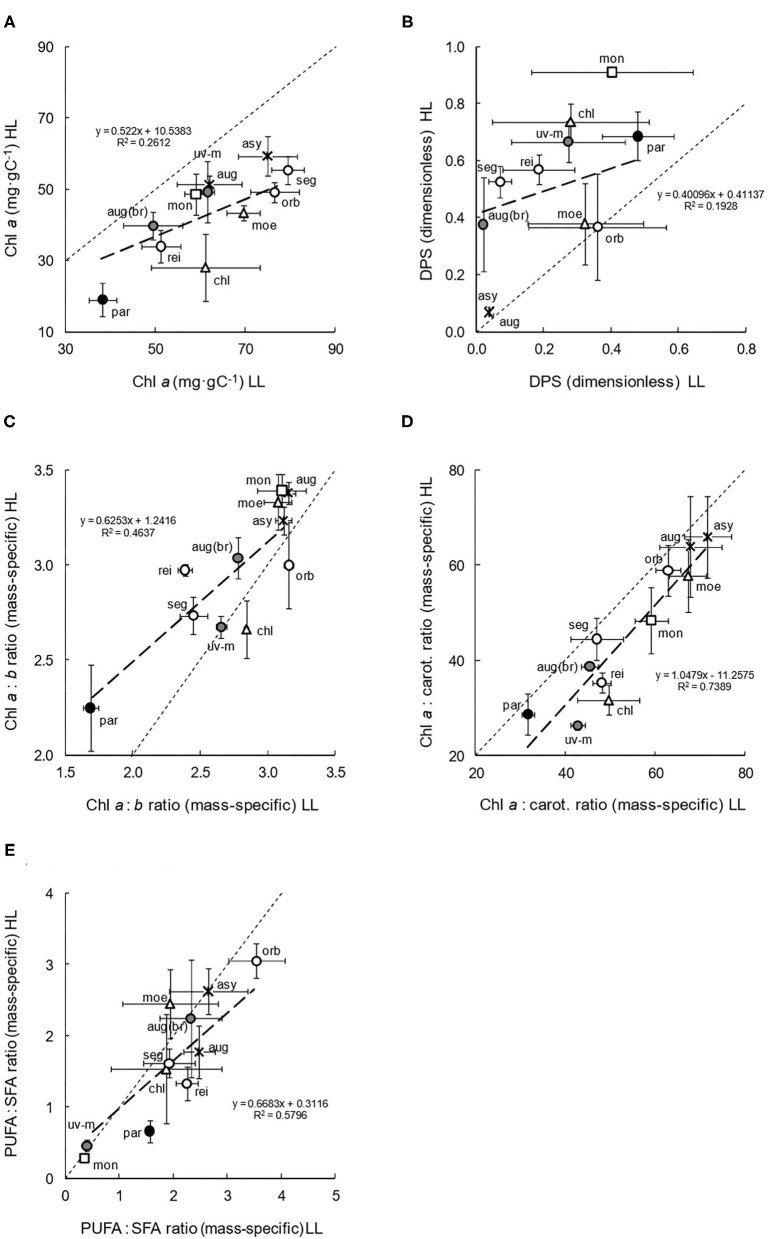
Compositional characteristics **(A–E)** of the 11 species of *Chlamydomonas* grown under conditions of low light (LL) and high light (HL). Mean values and standard errors are presented, with ratios in **(C–E)** being mass-specific (e.g., mg·gC^−1^). Thin dashed lines represent theoretical 1:1 (*x* = *y*) relationships between LL and HL adapted cultures, with thicker, linear regressions describing the observed relationships. The spatial spread of species data points gives an indication of intra-familial diversity, and their position above or below the 1:1 line indicates higher values in HL or LL culture irradiances, respectively, thereby suggesting plasticity. Symbols, shading and species abbreviations are as described in Figure 2. **(A)** Chl *a*. **(B)** DPS. **(C)** Chl *a*:*b* ratio. **(D)** Chl *a*:sum of carotenoid ratio. **(E)** PUFA:SFA ratio.

Across the 11 species, mean cell diameters ranged from 5 to 14 μm, and mean volumes ranged from 95 to 1,400 μm^3^. There were no clear relationships with habitat trophy, salinity, and clade; however, there was evidence of a very slight increase in mean cell volume (from 335.5 to 376.9 μm^3^) in the LL cultures (paired *t*-test: df = 50, *P* = 0.188). More intrinsic compositional results (Figures 3A–E) illustrated the diversity in pigment and fatty acid composition. Concentrations of chlorophyll *a* (Chl *a*) in relation to carbon content (Figure 3A), a basic indication of general photosynthetic and potential light absorbance capacity, were mostly higher in the LL cultures (paired *t*-test: df = 51, *P* < 0.001; Table 2). The adaptation was generally greatest in the eutrophic species and low in the Monadina clade. Overall, high Chl *a* concentrations were observed in oligotrophic species and low concentrations were found in *C. parkeae*. Concentrations of chlorophyll *b* (Chl *b*: data not presented) also increased in the LL cultures.

The de-epoxidation state (DPS, Figure 3B) of the xanthophyll-cycle components (whereby higher values are indicative of photoprotection and lower values, improved photosynthetic efficiency), significantly increased in the high light cultures (paired *t*-test: df = 51, *P* < 0.001: Table 2). The increase was particularly large in more nutrient-rich habitat, and marine and brackish, species. Overall, DPS at LL and HL was generally lower in oligotrophic species (as was total pigment concentration: data not presented) and higher in hypertrophic species and in the Monadina clade.

Chl *a*:Chl *b* ratios (Figure 3C), giving some indication of low-light adaptation, significantly decreased (and therefore shade-adaptation increased) in the low light cultures (paired *t*-test: df = 51, *P* < 0.001), although not significantly so in all the species (Table 2). Generally, this increased shade adaptation tended to be greatest in nutrient-rich habitat species. Overall, HL and LL ratios were slightly lower in the eutrophic and brackish species and much lower in the marine, *C. parkeae*. Shade adaptation was consequently higher in these species and lower in more oligotrophic species. Contrastingly, the Chl *a*: sum of carotenoid ratios (Figure 3D), which can be dually indicative of either low-light acclimation (*via* high ratios) or photoprotection (low ratios), significantly increased in the LL cultures (paired *t*-test: df = 51, *P* < 0.001), particularly in *C. reinhardtii, C. uva-maris, C. augustae* (br), and *C. chlorastera* (Table 2). In general, lower ratios (lesser LL acclimation/increased photoprotection) occurred in the marine and brackish species, with higher ratios (greater LL acclimation/decreased photoprotection) found in freshwater and particularly in the oligotrophic species.

Polyunsaturated to saturated fatty acids ratios (Figure 3E) also significantly increased in low light conditions (paired *t*-test: df = 38, *P* < 0.05), because of higher concentrations of polyunsaturated, ω-3 fatty acids, indicative of a potential increase in thylakoid membrane development and photoacclimation. Although not observed in all the species, significant increases in LL did occur in *C. monadina, C. reinhardtii*, and *C. parkeae* (Table 2). Additionally, there was no clear change in total fatty acid concentration with culture irradiance (paired *t*-test: df= 33, *P* = 0.702). However, stoichiometrically, the contribution and prevalence of individual fatty acids did vary with phylogenetic clade (data not presented). Overall, the freshwater species tended to show more compositional acclimation to light than the brackish and marine species (Table 2).

## Discussion

In this study we determined the nature and functional flexibility of behavioural, physiological, and compositional response across an environmentally cosmopolitan and ecologically important family of phytoflagellates. For the first time, we recorded a wide array of photoresponsive traits across the Chlamydomonadaceae, and an ecophysiological diversity and plasticity that may not only be phylogenetically derived but also attributable to the different typical habitats of species. Supporting the general paradigm in ecological theory, such multifarious strategies potentially allow for an exploitation of environmental conditions, optimising energy acquisition and ensuring ecological success.

### Behavioural Diversity and Acclimation

Behavioural response and movement ecology are often beguiling in their variety and complexity (Nathan et al., 2008), even in simple micro-organisms (Clegg et al., 2004b; Sengupta et al., 2017). Here, we observed similar, deceptively sophisticated, behaviour, and newly quantified the response of Chlamydomonadaceae from contrasting habitats, to ecologically representative gradients and variations in irradiance.

Strong behavioural photoresponses were displayed (Figure 1), directed and maintained by the sensory-mediated phototactic and photophobic swimming of cells [as in Clegg et al. (2003, 2012)], yet with differences in their magnitude, position, and strength of plasticity, due to habitat trophy, potentially facilitating a more precise and robust movement of cells in the often turbid, steeply attenuated light of more nutrient rich conditions. There was also a rarely observed [in microorganisms: cf. Bendesky and Bargmann (2011)], considerable inter-specific diversity, which may be explained by differences in the structure and functioning of intracellular sensory photoreceptive phytochromes (e.g., Gutu and Kehoe, 2012; Coesel et al., 2021), or the retention of behavioural traits (or syndromes: Sih et al., 2004) from environmental or physiological histories.

The unusual characteristic preferences for relatively low light widely displayed here were comparable with those of *C. moewusii* (Clegg et al., 2003), *C. acidophila* (Clegg et al., 2012), and even with the light at which *C. reinhardtii* aggregated in experimental water columns (Mellard et al., 2012). Although the phylogenetic origins of behaviour are difficult to ascertain, this photosynthetically paradoxical preference may be a canalised symplesiomorphic characteristic (a durable shared ancestral trait). It may help avoid harmful UV radiation (Häder et al., 2003) and photoinhibition (Richardson et al., 1983); and in combination with additional traits (e.g., Clegg et al., 2007, 2012), it may be energetically and physiologically beneficial. However, this predilection for low light was not universal. Interestingly, we discovered a considerable intra-familial difference in the behaviour of freshwater, compared to brackish and marine species; with the latter's broader preferences for higher light, potentially a consequence of increased turbulent mixing (*via* wind, wave, and tidal forces) and variable light (due to turbidity and surface-wave lensing) preventing a more precise behaviour being successfully applied in those environments.

We also investigated the plasticity of behavioural photoresponse (broadening its zoocentric scope: e.g., Buchanan et al., 2013) and found that six of the species (including all the brackish and marine species, whose broad preferences may render any acclimation redundant), showed no adaptation. This partially re-enforced existing research (e.g., Clegg et al., 2003) and emphasised the inherent stability of preferences (even in identical strains of *C. moewusii* subjected to historical differences in culture techniques). The robust behavioural stability and inertia observed here can often occur (cf. the cognitive resilience of birds, fish, and invertebrates: Buchanan et al., 2013), potentially dictated by physiological controls (Sih et al., 2004), and can be important for ecological fitness (e.g., Huey et al., 2003).

However, again, this lack of plasticity was not universal, as five of the eight freshwater species demonstrated a behavioural flexibility (similar to that recently observed in a single acidophile: Clegg et al., 2012) and a sophistication comparable with larger, more evolutionary complex, ecological type *K* species (e.g., *Ceratium furcoides*: Clegg et al., 2003). Such dynamic phenotypic adjustment may help buffer environmental perturbation. Indeed, species found in more challenging light (i.e., the more eutrophic species: Figure 1) or physico-chemical (e.g., Clegg et al., 2012) environments are generally more behaviourally labile; which would hypothetically assist competition for light in more turbid or productive ecosystems, while stability in oligotrophic species may help maintain position and guarantee access to nutrients typically found at depth. Behaviour can also change with ontogeny and nutrient status (Clegg et al., 2003, 2004a), and so this evolved divergence in different habitats is highly feasible (cf. microbial trade-off diversity: Gudelj et al., 2010).

In this study, we therefore observed both inter-species (longer term, genotypic) “photoadaptational” behavioural diversity, and intra-species (shorter term, phenotypic) “photoacclimational” behavioural plasticity, reaffirming the differentiation in Falkowski and Laroche (1991), and noted the complex influence of environmental and acclimation history, species, and even genetic strain (cf. Litchman and Klausmeier, 2008; Ajani et al., 2021). For example, although preference and acclimation may differ with the phylogenetic separation of species, typical habitat was highly important in dictating behaviour, as illustrated by the contrasting responses of geographically varied *C. augustae* ecotypes. Ecologically, the results help to expand the knowledge of phytoflagellate behaviour (and macroecological trait variability: Chown et al., 2004), identifying clear habitat-derived and species-specific variation in the Chlamydomonadaceae, which may be energetically beneficial in the face of environmental heterogeneity (e.g., Buchanan et al., 2013). However, in the search for a unifying paradigm of movement ecology (e.g., Nathan et al., 2008), this diversity and plasticity across just one family also highlights the widely appreciated complexities of behavioural ecology, from its potential internal motivation and interaction with physiological and compositional response, to its theoretical consequences for dispersal and foraging, and its broader ecological implications.

### Physiological Diversity and Acclimation

The physiological nature of photoresponse can vary with taxonomy and irradiance (e.g., Falkowski and Raven, 2007; Suggett et al., 2015). However, to date, aspects of its diversity and plasticity remain uncertain (e.g., Falkowski and Chen, 2003; Benning, 2015). In this study, we demonstrated a complex diversity and differential plasticity in growth, photosynthetic, and metabolic characteristics (Figure 2), noting a broad expression of physiotypes.

Although rates of growth generally fell within the range of comparable species (e.g., Wong et al., 2007), diversity was observed. Higher rates in eutrophic species (as in desmids: Spijkerman and Coesel, 1998) could be advantageous in light-limited ecosystems and lower rates in the Chloromonas clade could be due to life cycle nuances (e.g., Leya, 2013). More moderate rates in relation to other green algae (e.g., Schwaderer et al., 2011), indicative of a slight propensity for lower light, also decreased with irradiance; but not as significantly as in some genera (e.g., Richardson et al., 1983), potentially buffered by the auxiliary mixotrophic capacity of some Chlamydomonadaceae (e.g., Clegg et al., 2012). This plasticity was greatest in brackish, marine and eutrophic species, which may be beneficial in environments with greater light variability and deprivation (cf. Badyaev, 2005).

The photosynthetic capacity of species was less varied and typical of Chlamydomonads (e.g., Morita et al., 1999) and green algae in general (Richardson et al., 1983). Little plasticity was observed as P_max_ normalised to carbon is typically independent of irradiance (e.g., MacIntyre et al., 2002) but at low light can be optimised by concomitant metabolic, physiological, and compositional adjustments (Falkowski and Chen, 2003). Indeed, metabolic costs varied slightly across species, and quite low R_d_:P_max_ values (indicative of a slight low light propensity: cf. Gerloff-Elias et al., 2005b) also decreased slightly with culture irradiance in some species, a strategic flexibility that may facilitate energetic balancing at low light (e.g., Geider et al., 2009) and influence the depth limit of survival (e.g., Clegg et al., 2012).

Photosynthetic efficiency at low irradiance, α (in relation to carbon), also varied slightly with species; with higher values in some (e.g., *C. uva-maris*: susceptible to encounter more shaded conditions), potentially assisting survival at lower light. Alpha also increased considerably at lower culture irradiance, which (combined with a stable P_max_) signified photoregulatory optimisation of the effective absorption cross section of photosystem (PS) II (e.g., Falkowski and Chen, 2003). This plasticity was greatest in some eutrophic species (a possible artefact of α'*s* frequent co-dependence on light and nutrient availability: e.g., Cleveland and Perry, 1987), potentially influencing distribution and survival in more variable light environments (Ensminger et al., 2005). It may also be reinforced by additional adjustments to photosynthetic unit (antenna) size and number (indicated by P-I curve and pigment data), highlighting the complex integration of physiology and composition.

The compensation points for photosynthesis (important for critical depth and net energetics) also varied slightly with species and were towards the lower end of the green algal range (e.g., Striebel et al., 2009), suggesting some propensity for lower light (unusual for chlorophytes: Litchman and Klausmeier, 2008). This was reinforced by a widespread plasticity, which lowered E_c_ further under low light culture, even down to around 8 μmol photons m^−2^ s^−1^ in *C. uva-maris* (approaching deep chlorophyll maxima values: Geider et al., 1985). Saturation points for photosynthesis were slightly more varied across the species, but were also typical of the family (e.g., Clegg et al., 2012) and lower than many green algal values (Richardson et al., 1983). E_k_ also decreased slightly with culture irradiance in many species (expanding previous findings: Gerloff-Elias et al., 2005a), potentially optimising the quantum yield of photosynthesis. Interestingly, species that showed lesser adaptation of E_k_ tended to show a greater adaptation of E_c_, and vice-versa, indicative of antagonistic flexibility. Such complexities, and E_c_ and E_k_ in general, can influence distribution and have profound ecological implications (e.g., Clegg et al., 2012).

Overall, while reinforcing existing measurements, we also discovered a diverse physiology and strong physiological plasticity across the Chlamydomonadaceae, noting the potential effect of habitat and phylogeny. The contribution of environment was substantial, and the genetically defined physiological range also varied with class (cf. Richardson et al., 1983), clade (e.g., Chloromonas versus Monadina), and even ecotype (e.g., *C. augustae*). Ecologically, this complex diversity has broad ramifications and can influence niche adaptation and species distribution (e.g., Stomp et al., 2008; Suggett et al., 2015; Ajani et al., 2021). Similarly, physiological plasticity can reconcile environmental variation (particularly in light: e.g., MacIntyre et al., 2002), and can contribute to fitness and community dynamics (e.g., Litchman and Klausmeier, 2008). It may also combine (e.g., to energetically assist survival at low light: Clegg et al., 2012) with very closely entwined compositional photoresponses, which may ascribe the supramolecular, structural basis for many of these physiological adjustments.

### Compositional Diversity and Acclimation

Compositional photoresponses can also vary considerably with species and irradiance (e.g., Falkowski and Raven, 2007; Ajani et al., 2021), yet aspects again remain uncertain (e.g., Brunet and Lavaud, 2010). Here, we established a considerable compositional diversity, potentially attributable to habitat and phylogeny, and a complex compositional plasticity (Figure 3).

Stoichiometric and conformational architecture can be allometrically influenced (Litchman and Klausmeier, 2008); however, although we noted some diversity in (quite typical: cf. Fischer et al., 2006) cell sizes, and a slight increase in low light (that may lessen intracellular packaging constraints), the data were presented relative to carbon to avoid any distortion. Generally, photosynthetic and accessory pigment composition varied with species and light (cf. Falkowski and Chen, 2003). Total concentrations were higher in low light (as reaction and harvesting centres increased to modulate absorption and quantum yield) and largely comprised PSI and II associated complexes of Chl *a* and *b*, auxiliary xanthophylls, and carotenoids (as in terrestrial vascular plants).

There was a broad intra-familial diversity in Chl *a* concentration, spanning the typical range (e.g., Gerloff-Elias et al., 2005a). Concentrations were slightly higher in oligotrophic species (indicative of a high absorbance capacity or emergency nutrient store: e.g., Gallagher et al., 1984) and were lower in *C. parkeae*, because of its predominant yellow pigments [recorded here and in Sasa et al. (1992)], potentially derived from an ancestral adaptation to deeper, marine conditions. Indeed, such adaptive divergence may contribute to the substantial diversity noted here (even between *C. augustae* ecotypes). Chl *a* also increased in low light, most likely driven by the finely-tuneable redox poise of electron transport pathways (Falkowski and Chen, 2003). The increase varied with phylogeny [as in Richardson et al. (1983)] and was greater in the eutrophic species, again, potentially because of their susceptibility to shading. Increased antennal chlorophyll generally optimises energy acquisition under low light, although the interaction with physiology is complex (e.g., Falkowski and Chen, 2003), and the functional influence of other pigments must always be considered.

For example, de-epoxidation state (a measure of the modulation of energy flow by xanthophyll cycle pigments) varied across the family and was lower in the oligotrophic species. DPS can vary with species and their ecology (e.g., Dimier et al., 2009), and this lesser importance in clearer oligotrophic waters may not be photoprotectively counterintuitive (if overridden by a deep distribution). Of course, while high DPS can facilitate energy dissipation *via* non-photochemical quenching, lower DPS (derived from higher, non-quenching, violaxanthin concentrations) can benefit photosynthetic efficiency. Accordingly, DPS decreased slightly with culture irradiance in most species [e.g., as in Lohr and Wilhelm (1999)]. Although photoinhibition was unlikely (as β values remained unchanged), it can occur at quite low light (Richardson et al., 1983), often beginning slightly above E_k_ (which averaged 100 μmol photons m^−2^ s^−1^ here), and so an onset of photoprotection may explain the slight DPS increase at HL (354 μmol photons m^−2^ s^−1^), and even the atypical negative HL growth of *C. monadina*. Ecologically, this flexibility may impact distribution and survival in fluctuating light environments (Brunet and Lavaud, 2010), and can even be used to distinguish functional groups (Dimier et al., 2009).

Chl *a*:*b* ratios (indicative of shade adaptation) were typical of the family (e.g., Ruizzo et al., 1992), but varied considerably with species. Ratios were lower in the marine *C. parkeae* (because of its unusual composition) and in the eutrophic species (potentially in response to their more turbid environments), and even differed with ecotype (with higher ratios in the snow *C. augustae* suggestive of a tolerance for higher light). Ratios also decreased in several species under low light; as a complementary increase in Chl *b* signified a thylakoid and PSII enlargement that maximised absorption. Such plasticity is widespread, even in the Chlamydomonadaceae (e.g., Pineau et al., 2001). It can involve the catalysed conversion of Chl *a* to *b via* retrograde regulation (Hüner et al., 2012), or differential gene expression to give a phenotype rich in Chl *b* (Falkowski and Chen, 2003); and offers a viable mechanism of shade exploitation in challenging environmental conditions (e.g., Cruz et al., 2005).

Chl *a*:carotenoid ratios (indicative of low light acclimation or photoprotection) were comparable with and expanded existing data (e.g., Neale and Priscu, 1995), showing a tremendous intra-familial diversity. High concentrations of carotenoids (that dually serve as accessory light harvesters or quenching dissipaters) and lower ratios in marine, brackish, and more eutrophic species, most likely provide photoprotection in a more variable, water-current influenced light environment; whereas (analogous to DPS), low concentrations and higher ratios in oligotrophs may favour light exploitation in deep, nutrient-seeking (e.g., *C. subcaudata* in Neale and Priscu, 1995), and even snow species (e.g., C. *augustae* here, and *C. nivalis* in Remias et al., 2005); which may only accrue their conspicuous carotenoids at much higher light or as sessile cysts (e.g., Leya, 2013). Ratios also generally increased at low irradiance (as commonly observed: Falkowski and Raven, 2007), and this acclimation may interact with xanthophyll cycle processes (as a biochemical strategy: cf. Peers et al., 2009) to optimise light harvesting and photosystem ratios. Although observed in Chlamydomonadaceae (Peers et al., 2009), this plasticity is not universal (e.g., Gerloff-Elias et al., 2005a), and yet here we found that it was quite widely expressed, helping to ensure ecophysiological fitness in variable light (e.g., Hüner et al., 2012).

In addition to pigments, biochemical constituents such as fatty acids can also influence photosynthetic ecophysiology (e.g., Goss and Wilhelm, 2009), and, as integral components of photosystem membranes, their saturation can shape fluidity. Composition can vary with species and environment (e.g., Wacker et al., 2015). Indeed, here, total concentrations differed slightly and specific fatty acids varied considerably with phylogeny (because of a chemotaxonomic derivation of clades: Pröschold et al., 2001), but were generally comparable with similar strains (e.g., Wong et al., 2007). PUFA:SFA ratios also varied considerably with species and light; generally increasing at low light (as in other species: Spijkerman and Wacker, 2011), with higher polyunsaturated, ω-3 fatty acid concentrations (indicative of a greater thylakoid membrane development and functionality) assisting photoacclimation. Increases were significant in *C. monadina, C. reinhardtii*, and *C. parkeae* (potentially beneficial in their darker hypertrophic, soil, and marine environments), but not in *C. augustae*, even though they often occur in the cysts of snow species (e.g., Remias et al., 2005). Again, this flexibility can benefit photosynthesis and help overcome harsh environmental conditions (Spijkerman and Wacker, 2011).

Overall, the freshwater species tended to show more compositional photoacclimation than the brackish and marine (Table 2). Comparable with physiological response, this study reinforced and newly quantified many aspects of pigment and biochemical composition. It established a contrasting diversity and strong plasticity of compositional response across the Chlamydomonadaceae, often attributable to environmental and phylogenetic influences, and of functional ecological importance in addressing bio-optical variability (e.g., Brunet and Lavaud, 2010), attuning photosynthesis, and assisting survival (e.g., Falkowski and Chen, 2003).

### Functional Ecological Implications and General Conclusions

Few studies have attempted to identify the suite of responses expressed across an environmentally contrasting family of organisms; and in addition to the specific ecophysiological and ecological implications discussed above, the general diversity and complex plasticity we observed in the Chlamydomonadaceae is potentially of broader functional ecological importance. It may influence spatial and temporal distribution (e.g., Clegg et al., 2007); indeed, a relatively widespread behavioural and physiological predilection for quite low light generally supports their often-common occurrence in such conditions (e.g., Clegg et al., 2012). It can also have a crucial impact on growth and survival (e.g., Ensminger et al., 2005), with differences between species not only being energetically beneficial (e.g., Richardson et al., 1983), influencing fitness (e.g., Geider et al., 2009), and the ability of this family to thrive in many different environments (with applied implications: e.g., Smayda, 1997; Benning, 2015), but it may also shape niche processes, competition, succession, community composition, and ecosystem dynamics (e.g., Litchman and Klausmeier, 2008; Wang et al., 2020; Ajani et al., 2021).

In addition to helping resolve aspects of the broader functional contribution of photoresponsive traits to organism and species fitness, the results have highlighted the importance of diversity and plasticity (in behaviour, physiology, and composition) as part of a sophisticated response to environmental variation in light [while also helping to advance the understanding of ecophysiological predisposition in these species and providing an example of intra-familial differentiation: as encouraged by Ackerly et al. (2000)]. We observed adaptive differences across the Chlamydomonadaceae, and statistically significant trends in the dataset also indicated the occurrence of phenotypic plasticity-based (and potentially low-light beneficial) photoacclimation strategies. Such photobiological adaptation and acclimation often overlaps (Richardson et al., 1983). Moreover, the differential expression observed within and between species (Table 2) suggested that they may also combine in a complex manner. For example, physiology and composition may intricately interact (as illustrated by the antagonistic flexibility of E_c_ and E_k_) to form a dynamic regulatory network of transduced energetic response (Geider et al., 2009), and the differences expressed in the freshwater, brackish, and marine species could be of great significance. Future investigations will hopefully explore these interactions further; with an added emphasis on identifying the potential motivation behind the behavioural responses observed here, their relationship and balancing with other strategies, in different environments, and their implications for distribution.

Overall, this investigation therefore helped to determine the nature and extent of photoresponsive traits across an ecologically important family, emphasising their diversity, flexibility, complexity, and potential environmental derivation, while highlighting their broader ecological importance. This interdisciplinary study of behaviour, physiology, and composition has helped to improve the knowledge of phytoflagellate ecology and general ecophysiology. Having identified a rarely recorded phenotypic diversity and sophisticated plasticity, this study advocates the importance of an integrated approach for enhancing the understanding of the relationship between organisms and their environments, and the environmentally contrasting nature of the study species means that the findings could be broadly applicable across the aquatic and terrestrial ecology divide.

## Data Availability Statement

The raw data supporting the conclusions of this article will be made available by the authors, without undue reservation.

## Author Contributions

MC, ES, and AW conceived the ideas, designed the methodology for this study, were responsible for behavioural, photosynthetic and fatty acid measurements, respectively, and performed all data processing and analyses. MC wrote the manuscript with substantial contributions throughout from ES and AW. All authors gave final approval for publication.

## Conflict of Interest

The authors declare that the research was conducted in the absence of any commercial or financial relationships that could be construed as a potential conflict of interest.

## Publisher's Note

All claims expressed in this article are solely those of the authors and do not necessarily represent those of their affiliated organizations, or those of the publisher, the editors and the reviewers. Any product that may be evaluated in this article, or claim that may be made by its manufacturer, is not guaranteed or endorsed by the publisher.
